# Matrine Reverses the Warburg Effect and Suppresses Colon Cancer Cell Growth *via* Negatively Regulating HIF-1α

**DOI:** 10.3389/fphar.2019.01437

**Published:** 2019-11-28

**Authors:** Xiaoting Hong, Linhai Zhong, Yurou Xie, Kaifeng Zheng, Jinglong Pang, Yesen Li, Yifan Yang, Xiaolin Xu, Panying Mi, Hanwei Cao, Wenqing Zhang, Tianhui Hu, Gang Song, Daxuan Wang, Yan-yan Zhan

**Affiliations:** ^1^Cancer Research Center, School of Medicine, Xiamen University, Xiamen, China; ^2^Department of Basic Medicine, School of Medicine, Xiamen University, Xiamen, China; ^3^Department of Nuclear Medicine and Minnan PET Center, Xiamen Cancer Hospital, The First Affiliated Hospital of Xiamen University, Xiamen, China; ^4^Department of Respiratory Medicine, Fujian Provincial Hospital, Fuzhou, China

**Keywords:** matrine, HIF-1α, Warburg effect, colon cancer, glucose metabolism

## Abstract

The Warburg effect is a peculiar feature of cancer’s metabolism, which is an attractive therapeutic target that could aim tumor cells while sparing normal tissue. Matrine is an alkaloid extracted from the herb root of a traditional Chinese medicine, *Sophora flavescens* Ait. Matrine has been reported to have selective cytotoxicity toward cancer cells but with elusive mechanisms. Here, we reported that matrine was able to reverse the Warburg effect (inhibiting glucose uptake and lactate production) and suppress the growth of human colon cancer cells *in vitro and in vivo*. Mechanistically, we revealed that matrine significantly decreased the messenger RNA (mRNA) and protein expression of HIF-1α, a critical transcription factor in reprogramming cancer metabolism toward the Warburg effect. As a result, the expression levels of GLUT1, HK2, and LDHA, the downstream targets of HIF-1α in regulating glucose metabolism, were dramatically inhibited by matrine. Moreover, this inhibitory effect of matrine was significantly attenuated when HIF-1α was knocked down or exogenous overexpressed in colon cancer cells. Together, our results revealed that matrine inhibits colon cancer cell growth *via* suppression of HIF-1α expression and its downstream regulation of Warburg effect. Matrine could be further developed as an antitumor agent targeting the HIF-1α-mediated Warburg effect for colon cancer treatment.

## Introduction

Cancer cells have remarkably increased metabolic requirements in comparison to normal cells. In order to support this demand, cancer cells consume much more glucose and produce lactic acid rather than catabolizing glucose by the tricarboxylic acid cycle. By this way, they would be able to consume additional nutrients and divert those nutrients into macromolecular synthesis pathways to support their rapid proliferation (Vander Heiden et al., 2009). This phenomenon is termed as the “Warburg effect,” as first described by Otto Warburg in 1920s (Warburg, 1956).

Hypoxia-induced factor 1 (HIF-1) is a heterodimeric protein composed of two subunits, HIF-1α and HIF-1β. HIF-1α is accurately regulated by oxygen concentration while HIF-1β is constitutively expressed in cells. Under normoxic conditions, HIF-1α is hydroxylated on proline residue 402 and/or 564, which is required for binding of the von Hippel–Lindau (VHL) protein, the recognition subunit of an E3 ubiquitin ligase that targets HIF-1α for proteasomal degradation. Upregulation of HIF-1α is usually detected in cancer cells, which is caused by the hypoxic condition in the solid tumor as well as the activation of some oncogene (Denko, 2008; Masoud and Li, 2015). As a critical mediator of the Warburg effect, HIF-1 stimulates the expression and activation of glycolytic enzymes, thereby supporting the Warburg effect in human cancers (Lu et al., 2002; Courtnay et al., 2015). Targeting HIF-1α to inhibit the Warburg effect has emerged as a promising anticancer therapeutic strategy (Semenza, 2003; Chen et al., 2007).

Colon cancer is one of the leading causes of cancer death in the developed world. Surgery is the main treatment for colon cancer, while radiotherapy and chemotherapy are also important to improve prognosis. However, chemotherapy resistance is still a big challenge for treatment. Discovery of novel drugs for colon cancer treatment would be the way to overcome this dilemma. Matrine is an active component extracted from the herb root of a traditional Chinese medicine, *Sophora flavescens Ait*. Matrine has long been used for the treatment of viral hepatitis, liver cirrhosis, and skin inflammatory (Liu et al., 2003). Recently, the antitumor effect of matrine has provoked considerable interest, manifested as inhibiting cell proliferation, accelerating apoptosis, inducing cell cycle arrest and differentiation, suppressing metastasis, invasion, and angiogenesis in a variety of malignant cells (Zhang et al., 2010; Guo et al., 2013; Liu et al., 2014; Ma et al., 2015; Xu et al., 2017), including colon cancer (Chang et al., 2013; Zhang et al., 2014; Gu et al., 2018). However, the precise molecular target of matrine is still mostly unknown. In this article, we found that matrine inhibited the transcription of HIF-1α, thereby reversing the Warburg effect (inhibiting glucose uptake and lactate production) and suppressing cell growth in human colon cancer cells *in vitro* and *in vivo*. These results provide a theoretical basis for the application of matrine as an effective anticancer drug for colon cancer.

## Materials and Methods

### Antibodies and Reagents

Matrine was purchased from Sigma-Aldrich (Catalog No. M5319, purity above 97%, Saint Louis, MO, USA). Antibody for HIF-1α was purchased from BD Bioscience (Catalog No. 610958, San Diego, CA, USA). 2-(N-(7-Nitrobenz-2-oxa-1,3-diazol-4-yl)Amino)-2-deoxyglucose (2-NBDG) was purchased from Thermos Fisher Scientific (Eugene, OR, USA). All other reagents were from Sigma-Aldrich (Shanghai, China) unless stated otherwise.

### Cell Lines and Cell Culture

Human colon cancer cell lines HCT116 and SW620 were obtained from Cell Bank of Chinese Academy of Sciences (Shanghai, China). All the cell lines were authenticated by SNP and short tandem repeat analyses by the provider. HCT116 cells were cultured in McCoy’s 5A medium. SW620 cells were cultured in modified Eagle’s medium. All the medium was supplemented with 10% fetal bovine serum (HyClone, Logan, UT, USA), 100 µg/ml streptomycin, and 100 U/ml penicillin (Life Technologies, Carlsbad, CA, USA).

### 3-(4,5-Dimethylthiazol-2-yl)-2,5-diphenyltetrazolium Bromide Assay

Cell viability was detected using 3-(4,5-dimethylthiazol-2-yl)-2,5-diphenyltetrazolium bromide (MTT) assay. Cells were seeded at 1 × 10^4^ cells/well in 96-well plates and then treated with matrine as indicated. After treatment, 50 µl of 5 mg/ml MTT solution was added to each well and incubated at 37°C for 4 h. The formazan crystals formed were then dissolved in 150 µl DMSO, and the absorbance of the solution was then obtained on a microplate reader at λ570 nm. Results were presented as the percentage loss of cell viability compared with control.

### Colony-Forming Assay

Cells were seeded in six-well plate. After 24 h, cells were treated with matrine for 12 h. After treatment, cells were washed with PBS, harvested by trypsinization, counted, and seeded into six-well dishes at 1,000 cells/well. The cells were incubated for another 10 days, then fixed and stained with 1% crystal violet in ethanol. Colonies containing 50 or more cells were scored. Colony formation was normalized to the colony forming efficiency of the non-drug-treated group to calculate the surviving fraction.

### Tumor Xenograft in Nude Mice

The animal experiments were approved by the Committee on the Ethics of Animal Experiments of Xiamen University. Female Balb/c nude mice aged 4–6 weeks were inoculated with 2 × 10^6^ HCT116 cells by subcutaneous injection into the right flank. When tumors reached an average volume > 120 mm^3^, mice were administrated with 100 µl saline (control group) or 0.32 mmol/kg matrine every day by intraperitoneal injection. The tumor volumes were determined according to the equation (volume = length×width^2^) and recorded every day.

### Glucose Uptake Assay

Glucose uptake of cells was measured by 2-NBDG uptake as described by Zou et al. (2005). Briefly, cells were seeded in 12-well plates at a density of 70–80% and treated with matrine as indicated. After treatment, cells were harvested and resuspended in Krebs-Ringer’s HEPES Buffer (KRB) solution, and incubated with 100 nmol/L 2-NBDG at 37°C in 5% CO_2_ for 30 min. The 2-NBDG uptake reaction was stopped by removing the incubation medium and washing the cells twice with pre-cold phosphate buffered saline (PBS). One microgram per milliliter of propidium iodide (PI) solution was used to distinguish dead cells. The fluorescence was then measured by flow cytometry.

### *In Vivo* Glucose Uptake Assay

Nude mice bearing SW620 colon cancer cells xenografts were administrated with 3.7–7.4 MBq (100–200 µCi) of [18F]-FDG *via* the tail vein. After injection, the mice were anesthetized with isoflurane (5% for induction and 2% for maintenance) to minimize mice movement and minimize muscle uptake of glucose. 40 min after injection, a whole body CT scan (6 min) were performed following with microPET images (5 min). The microPET and CT images were generated separately and then fused using Inveon Research Workplace version 4.1 (Siemens Medical Solutions, Inc., USA). The images were reconstructed using ordered subset expectation maximization with three-dimensional resolution recovery (OSEM 3D) with CT-based attenuation correction and scatter correction. For data analysis, the region of interests (ROI) were manually drawn and covered the whole tumor on the CT images. This ROI was copied to the corresponding PET images. The percentage injected dose per gram tissue (%ID/g) of the tumor in the ROIs were recorded as the uptake of glucose.

### Lactate Production Assay

Cells were seeded in six-well plates at a density of 70–80% and treated with matrine as indicated. After treatment, the culture medium of each well was carefully collected. The lactate concentration in the medium was measured using the Lactate Colorimetric Assay Kit II from BioVision, Inc. (Milpitas, CA) according to manufacturer’s instruction. Briefly, samples were mixed with the provided reaction reagent and incubated for 30 min in room temperature, and the optical density (OD) value was measured at 450 nm. The measured OD values of the samples were compared with that of the standard lactate control to calculate the concentration of lactate. The levels of lactate were then normalized to the non-drug-treated group.

### Quantitative Real-Time Polymerase Chain Reaction

Cells were seeded in six-well plates at a density of 70–80% and treated with matrine as indicated. Total RNA was isolated using TRIzol reagent (Takara, Dalian, China). RNA was treated with DNase to remove genomic DNA and was reverse-transcribed into cDNA using Primescript^™^ RT reagent kit (Takara, Dalian, China) according to the manufacturer’s protocol. Real-time quantitative polymerase chain reaction (PCR) was carried out with the SYBR Green I fluorescent dye method (SYBR^®^ Premix Ex TaqTM II, Takara, Dalian, China) and the StepOnePlus real-time PCR apparatus (Applied Biosystems, Australia). The sequences of primers used are as follows: forward: 5′-TATTGCACTGCACAGGCCACATTC-3′ and reverse: 5′-TGATGGGTGAGGAATGGGTTCACA-3′ for HIF-1α; forward: 5′-GGCATTGATGACTCCAGTGTT-3′ and reverse: 5′-ATGGAGCCCA GCAGCAA-3′ for GLUT1; forward: 5′-TCACGGAGCTCAACCATGAC-3′ and reverse: 5′-CTG CAGTAGGGTGAGTGGTG -3′ for HK2; forward: 5′-GCCCGACGTGCATTCCCGATTCCTT-3′ and reverse: 5′-GAC GGCTTTCTCCCTCTTGCTGACG-3′ for LDHA; forward: 5′-CGTGTACTACAATGAGGCTGC-3′ and reverse: 5′-CTGGTCTGAAGATCTGGCCG-3′ for β-tubulin. The amplification specificity was checked by melting curve analysis. The relative expression of messenger (mRNA) was calculated using the 2^−△△Ct^ method through normalizing to mRNA of β-tubulin. The fold change of matrine treated group was calculated by normalizing to control (non-drug treated) group.

### Western Blotting

Cells at 60–80% confluence were washed with PBS and lysed directly into SDS–PAGE loading buffer. Twenty micrograms of protein was analyzed by SDS–PAGE and transferred to PVDF membrane. Primary antibodies (HIF-1α BD Bioscience 610958, HIF-1β CST-3414) were used at 1:1,000 in 5% milk in Tris-buffered saline with 0.05% Tween-20. Immunopositive bands were visualized by Amersham ECL^™^ Plus Western Blotting Detection Kit (GE Healthcare, Piscataway, NJ, USA).

### Immunohistochemistry

Standardized immunohistochemical stainings were performed on formalin-fixed paraffin-embedded (FFPE) xenograft tumor tissue. Five-micrometer-thick sections were placed on coated glass slides, deparaffinized, and rehydrated and then subjected to high-pressure antigen retrieval in a pressure cooker for 3 min in preheated 10 mmol/L sodium citrate buffer (pH 6.0). Endogenous peroxidase activity was blocked by incubation in 3% hydrogen peroxide for 10 min, and nonspecific staining was eliminated by incubating the sections with 10% normal goat serum for 15 min at room temperature. The sections were incubated with the primary antibody (HIF-1α BD Bioscience 610958, GLUT1 Abcam ab115730, LDHA CST-3582, HK2 Abcam ab209847) at 4°C overnight. Sections were incubated with diluted biotinylated secondary antibody for 10 min and then incubated with the avidin-biotin-peroxidase complex for another 10 min with repeated washing steps. Staining was visualized using 3,3’-diaminobenzidine solution (Maxim, Fuzhou, China). Sections were then counterstained with hematoxylin after dehydration and scanned by Motic digital slide scanner.

### Ribonucleic Acid Interference and HIF-1α Overexpression

RNA interference of HIF-1α expression was performed by stable cell lines expressing short hairpin RNA (shRNA). GV112 lentiviruses vectors (GeneChem, Shanghai, China) were cloned with shRNA targeting HIF-1α (target sequence: GCTGGAGACACAATCATAT and CTCTTTGTGGTTGGATCTA). Lentivirus was packaged and infected cells. Stable cell lines were selected by puromycin and the knockdown effect was confirmed by qPCR and Western blotting. Human HIF-1A cDNA was cloned into pCMV5 vectors and were transfected into HCT116 and SW620 cells by Lipofectamine 2000. Forty-eight hours of after transfection, the overexpression effect was confirmed by qPCR and Western blotting, and cytotoxicity assay was performed as indicated.

### Statistical Analysis

The SigmaPlot version 11.0 software package was used for statistical analysis. The results are presented as mean ± standard error (SEM). Data were analyzed by one-way analysis of variance (ANOVA) or Student’s *t*-test. *P* < 0.05 was considered significant.

## Results

### Matrine Inhibits Colon Cancer Cell Growth

**Figure 1:**
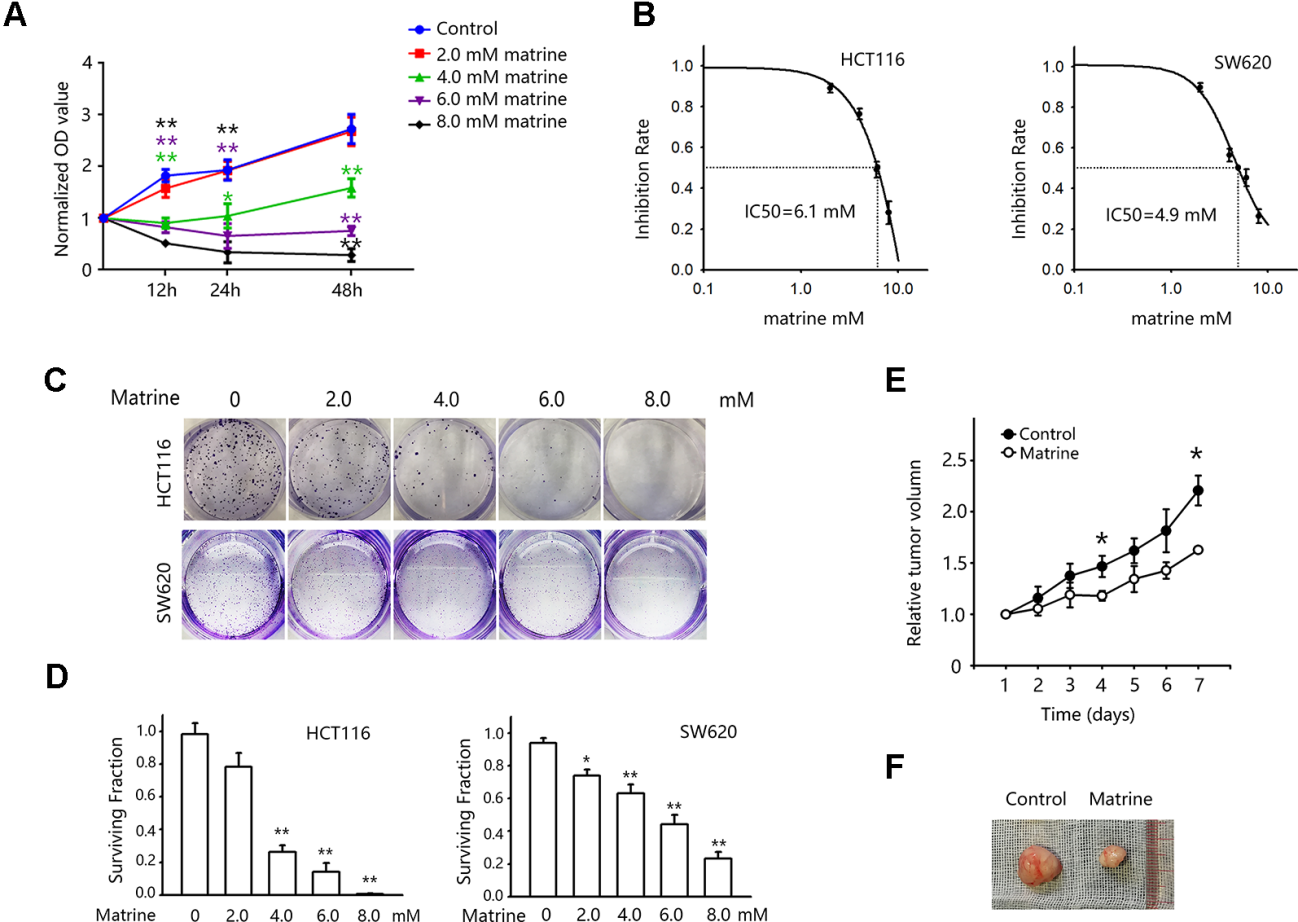
Matrine inhibited the growth of colon cancer cells *in vitro and in vivo*. **(A)** 3-(4,5-Dimethylthiazol-2-yl)-2,5-diphenyltetrazolium bromide (MTT) assay to examine cell viability after matrine treatment. Cells were treated with different concentrations of matrine as indicated for 12∼48 h. Data were expressed as mean ± SEM, n = 6. **, *P* < 0.01. **(B)** MTT assay to examine the IC50 of matrine on HCT116 and SW620 cells for 12 h of treatment. Data are expressed as mean ± SEM, n = 6. **(C)** Representative pictures of colony forming assay to show the growth inhibition effect of different concentrations of matrine on colon cancer cells. **(D)** Surviving fraction of colony forming assay. Data were expressed as mean ± SEM, n = 3. *, *P* < 0.05, **, P < 0.01. **(E)** Growth curve of colon cancer xenograft tumor with/without matrine treatment. Data were expressed as mean ± SEM, n = 5. *, *P* < 0.05. **(F)** Representative pictures of colon cancer xenograft tumors with/without matrine treatment.

To validate the anticancer effect of matrine on colon cancer cells, we first examine the impact of matrine on HCT116 cells growth in different concentrations and treating time. As shown in **Figure 1A**, treatment of 4.0 mM matrine has significant growth inhibitory effect on HCT116 cells (green line). When the concentrations increased to 6.0 mM or above (8.0 mM), matrine shows significant cytotoxicity (**Figure 1A**, purple and black lines). We then calculated the IC50 of matrine for 12-h treatment in two different colon cancer cell lines (HCT116 and SW620). The IC50 of matrine in HCT116 cells is 6.1 mM, while SW620 cells are slightly more sensitive with IC50 of 4.9 mM (**Figure 1B**). The growth inhibitory effects were also validated by colony forming assay. As shown in **Figures 1C, D**, the surviving fraction decreased with increasing concentrations of matrine. These results suggest that matrine has an efficient anticancer effect on colon cancer cells.

To further validate our finding of matrine in tumor growth inhibition *in vivo*, nude mice harboring colon cancer cells xenograft were used. Mice were inoculated with HCT116 cells, and treatments were given when tumors reached an average volume of 120 mm^3^. Mice were randomly divided into control group (saline injection) and matrine group (0.32 mmol/kg, i.p/day). And the tumor volumes were recorded every day for 7 days. **Figures 1E, F** show the effect of matrine on the growth of HCT116 tumors in nude mice. There was a significant difference in the average tumor volumes between the control and matrine groups after 7 days of treatment.

### Matrine Suppresses the Warburg Effects in Colon Cancer Cells

**Figure 2:**
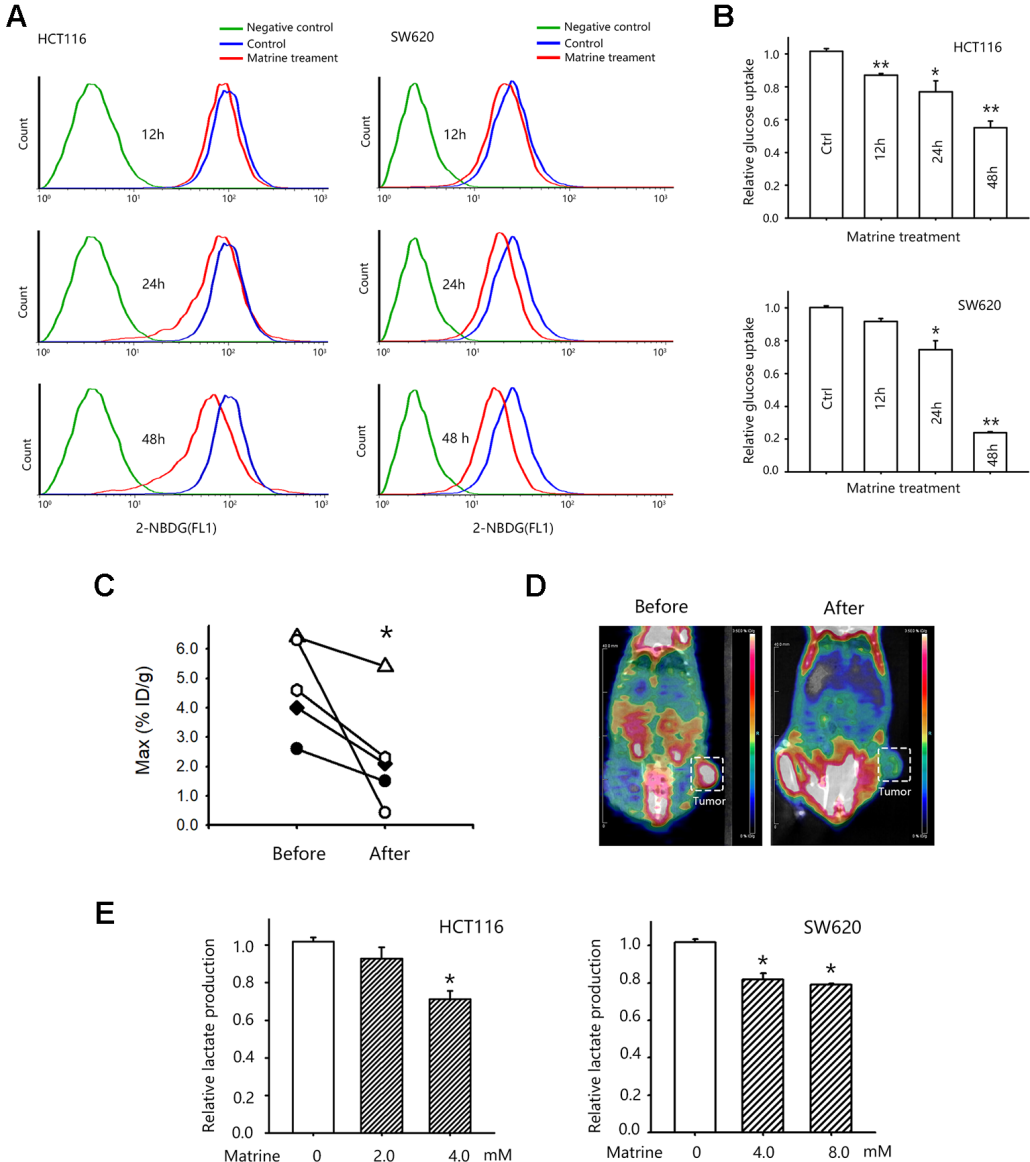
Matrine inhibited glucose uptake of colon cancer cells *in vitro and in vivo*. **(A)** Representative pictures of flow cytometry for accessing glucose uptake in HCT116 and SW620 cells. **(B)** Quantified data of glucose uptake determined by flow cytometry in HCT116 and SW620 cells after matrine treatment. Data were expressed as mean ± SEM, n = 3. *, *P* < 0.05, **, *P* < 0.01. **(C)** Quantified data of glucose uptake determined by microPET/CT. *, *P* < 0.05, paired Wilcoxon test. **(D)** Representative microPET/CT images of nude mice bearing subcutaneous SW620 tumor, which were acquired at 40 min after i.v. injection of [18F]-FDG. **(E)** Lactate production of HCT116 and SW620 cells with the treatment of matrine. Data were expressed as mean ± SEM, n = 4∼6. *, *P* < 0.05.

Warburg effect is one of the ways that cancer cells could sustain its rapid growth by facilitating the uptake of nutrition for biomass (Vander Heiden et al., 2009). Warburg effect is manifested as the increased glucose uptake and enhanced the production of lactate for generating ATP (Chen et al., 2007). To investigate whether the growth inhibitory effect of matrine is related to this metabolic change, we first detected the glucose uptake in HCT116 and SW620 cells after matrine treatment. **Figure 2A** showed that treatment with matrine reduced glucose uptake in these two cell lines. Forty-eight hours of treatments with 4.0 mM matrine reduced glucose uptake of HCT116 by 42% and SW620 by about 80%, respectively (**Figure 2B**). We next examined these effects *in vivo* by [18F]-FDG following microPET imagine. Nude mice harboring SW620 cells xenograft were first scanned for the glucose uptake in tumor xenograft. Then mice were treated with matrine for 0.32 mmol/kg. Twenty-four hours later, mice were re-scanned to detect the effect of matrine on glucose uptake. As shown in **Figures 2C, D**, matrine treatment substantially decreased glucose uptake in tumors. In the most sensitive mice, matrine inhibited glucose uptake by almost 80%. These data indicated that matrine inhibits glucose consumption in colon cancer cells *in vitro* and *in vivo*.

We then examined the effect of matrine on aerobic glycolysis by detecting the lactate production in matrine-treated colon cancer cells. We observed that matrine inhibited lactate production in HCT116 and SW620 cells. Treatments with 4.0 mM matrine for 24 h reduced lactate production of HCT116 by around 28% and SW620 by about 20%, respectively (**Figure 2E**). Taken together, our results suggested that matrine interrupted colon cancer metabolism, i.e., matrine inhibited the Warburg effects by suppressing glucose consumption and lactate production in colon cancer.

### Matrine Inhibits the Transcription and Protein Expression of HIF-1α

**Figure 3:**
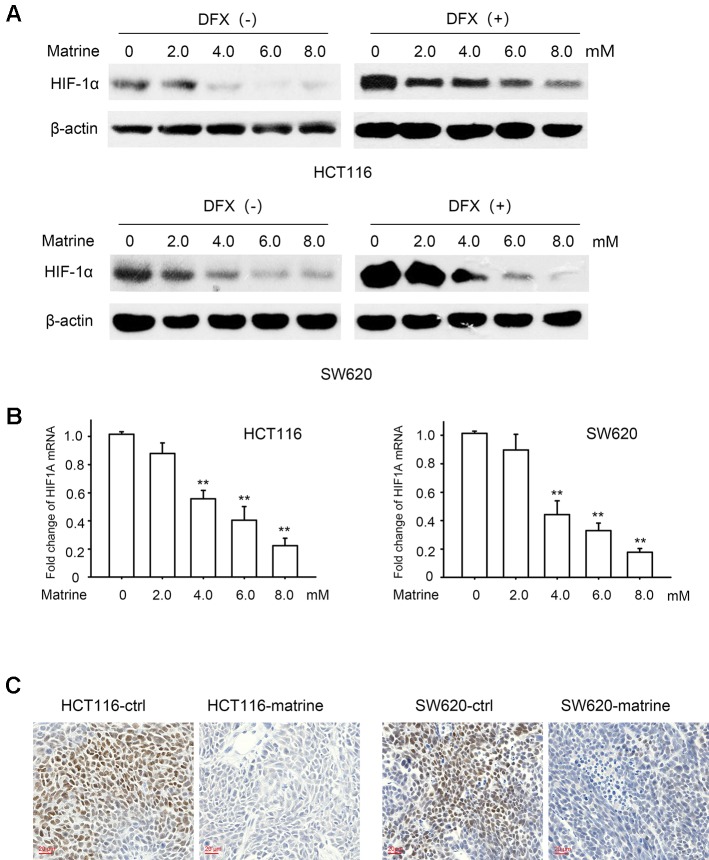
Matrine suppressed messenger RNA (mRNA) transcription and protein expression of HIF-1α. **(A)** Western blotting shows the protein expression of HIF-1α in HCT116 and SW620 cells with 12 h treatment of 0∼8.0 mM matrine together with or without 100 μM desferrioxamine treatment. **(B)** HIF-1α mRNA level as quantified by quantitative real-time-PCR in HCT116 and SW620 cells with the treatment of 0∼8.0 mM matrine for 12 h. Data were expressed as mean ± SEM, n = 6. **, *P* < 0.01. **(C)** Immunohistochemistry showing the expression of HIF-1α in mouse xenograft tumors.

We next investigated the potential mechanisms governing the glycolytic phenotype changing associated with matrine treatment. HIF-1 pathway is a vital regulator of the Warburg effect and upregulated HIF-1α expression has often been seen in many cancer cells. In the two colon cancer cell lines we tested, we found a detectable HIF-1α protein expression in normoxic condition (**Figure 3A**, first lands), which suggested that there is residual HIF-1 activity in these colon cancer cells. Treatment with different concentrations of matrine largely reduced HIF-1α protein expression (**Figure 3A**, left panel) in normoxic condition.

Protein degradation is known to be a vital regulatory process for cellular HIF-1α. To investigate whether the effect of matrine on HIF-1α is related to protein degradation, we treated cells with desferrioxamine (DFX), an iron chelator, which is known to mimic hypoxia condition by blocking HIF-1α degradation (Jaakkola et al., 2001). Matrine reduced the protein level of HIF-1α even when the degradation was blocked by DFX (**Figure 3A** right panel). These results suggest that the negative regulation of matrine on HIF-1α proteins may be independent of the protein degradation.

We then evaluated matrine’s effect on the mRNA levels of HIF-1α. As indicated in **Figure 3B**, with the treatment of matrine for 12 h, the mRNA level of HIF-1α decreased in a dose-dependent manner in both HCT116 and SW620 cells. Thus, our data indicated that matrine inhibits HIF-1α mRNA expression, thereby reducing the protein level of HIF-1α. We also examined the effect of matrine on HIF-1β, which is the other component of heterodimeric HIF-1. However, matrine did not affect the mRNA and protein expression levels of HIF-1β in the two colon cancer cells (**Figure S1**).

To validate the inhibitory effect of HIF-1α by matrine *in vivo*, the xenograft tumors were isolated and immunohistochemistry was performed to examine HIF-1α expression. As shown in **Figure 3C**, treatment with matrine could reduce the protein expression of HIF-1α in tumor xenograft samples.

### Matrine Inhibits Glucose Metabolism-Related Gene Transcription in Colon Cancer Cells

**Figure 4:**
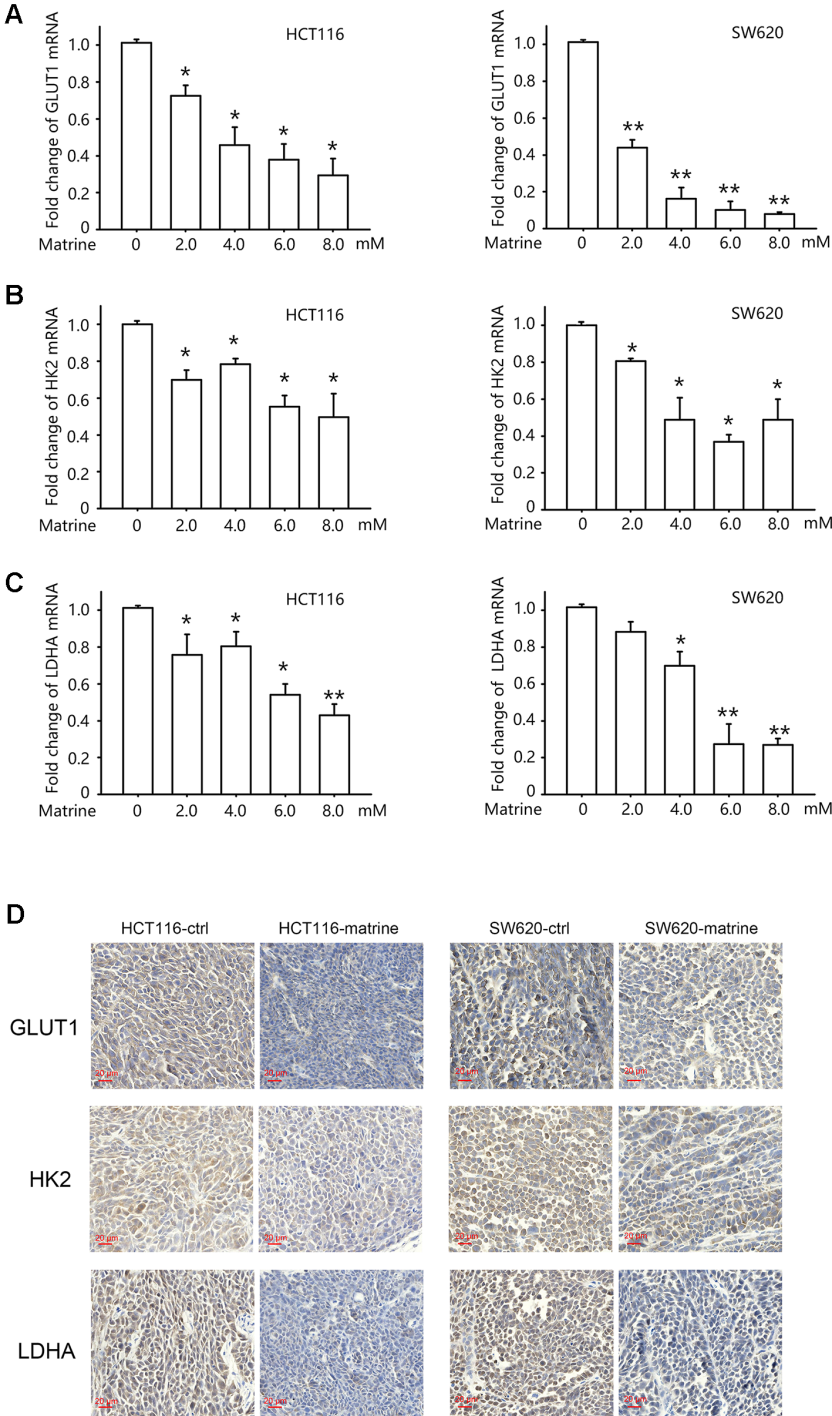
Matrine inhibited the transcription of glucose metabolism-related genes in colon cancer cells. **(A–C)** Quantitative real-time-PCR analysis of GLUT1, HK2, and LDHA in HCT116 and SW620 cells following treatment with 0∼8.0 mM matrine for 12 h. Data were expressed as mean ± SEM, n = 4∼6. *, *P* < 0.05. **, *P* < 0.01. **(D)** Immunohistochemistry showing the expression of GLUT1, HK2, and LDHA in mouse xenograft tumors.

The role of HIF-1α in reprogramming cancer metabolism toward the Warburg effect depends on its transcription activity, i.e., the activation of HIF-1 pathway would enhance the transcription of glucose metabolism gene such as glucose transporter 1 (GLUT1), hexokinases2 (HK2), and lactate dehydrogenase A (LDHA) (Semenza, 2003). GLUT1 facilitates the transport of glucose across the plasma membranes. Hexokinase 2 phosphorylates glucose to produce glucose-6-phosphate (G6P), the first step in most glucose metabolism pathways. LDHA catalyzes the synthesis of (S)-lactate from pyruvate. To examine the effect of matrine on these downstream effects of HIF-1α, we detected the mRNA level of these glucose metabolism-related enzymes after matrine treatment. As shown in **Figure 4**, 12 h treatment with matrine dose-dependently reduced the transcription of GLUT1, HK2, and LDHA in both HCT116 and SW620 cells.

We then examine protein levels of these three genes in tumor xenograft sample by immunohistochemistry. As shown in **Figure 4D**, treatment with matrine could reduce the protein levels of GLUT1, HK2, and LDHA.

### The Inhibitory Effect of Matrine on Warburg Effect and Cell Growth Is Attenuated by HIF-1α Knockdown or Overexpression in Colon Cancer Cells

**Figure 5:**
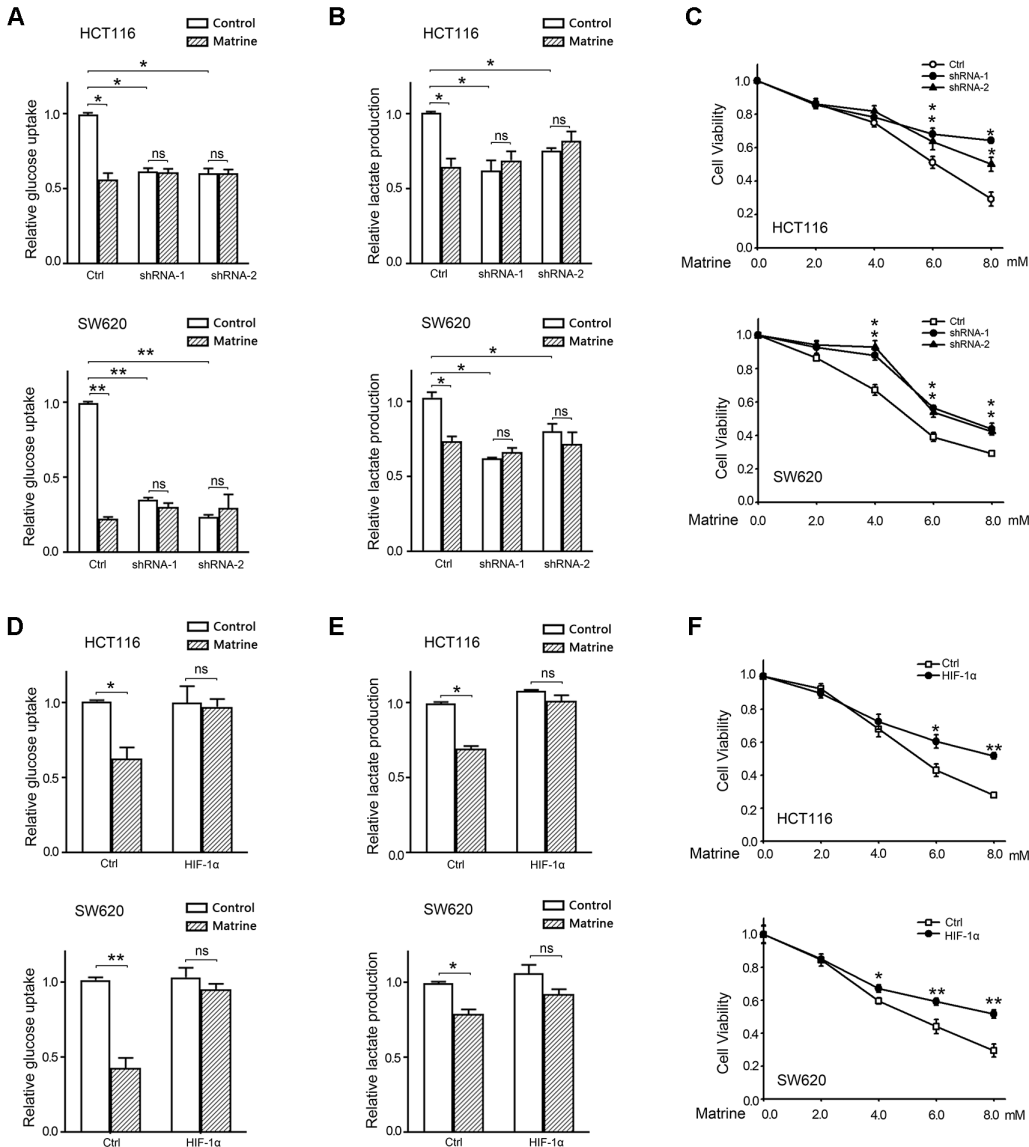
The growth inhibitory effect of matrine is attenuated by knockdown or overexpression of HIF-1α. **(A)** Quantified data of glucose uptake determined by flow cytometry in HCT116 and SW620 cells after 4.0 mM matrine treatment for 48 h. Data were expressed as mean ± SEM, n = 3. *, P < 0.05, **, *P* < 0.01, ns: not significant. **(B)** Lactate production of HCT116 and SW620 cells with the 4.0 mM matrine treatment for 24 h. Data were expressed as mean ± SEM, n = 3. *, P < 0.05, ns: not significant. **(C)** Cell viability accessed by 3-(4,5-dimethylthiazol-2-yl)-2,5-diphenyltetrazolium bromide (MTT) assay after treatment with 0∼8.0 mM matrine for 12 h. Data were expressed as mean ± SEM, n = 3∼5, *P < 0.05. **(D)** Quantified data of glucose uptake determined by flow cytometry in HCT116 and SW620 cells after 4.0 mM matrine treatment for 48 h. Data were expressed as mean ± SEM, n = 3. *, P < 0.05, ns: not significant. **(E)** Lactate production of HCT116 and SW620 cells with the 4.0 mM matrine treatment for 24 h. Data were expressed as mean ± SEM, n = 3. *, P < 0.05, ns: not significant. **(F)** Cell viability accessed by MTT assay after treatment with 0∼8.0 mM matrine for 12 h. Data were expressed as mean ± SEM, n = 3∼5, *P < 0.05, **, *P* < 0.01.

To investigate whether the effect of matrine on reversing Warburg effect and inhibiting colon cancer growth is due to the inhibition on HIF-1α, we used shRNA to knockdown endogenous HIF-1α expression in HCT116 and SW620 cells. Two different shRNA targeting HIF-1α were constructed, and stable HCT116 and SW620 cell lines expressing shRNA were established. Quantitative real-time PCR (qRT-PCR) and Western blotting conferred the knockdown effect of these shRNAs (**Figures S2A, B**). Glucose uptake and the production of lactate were analyzed in HIF-1α knockdown cells. As shown in **Figures 5A, B**, knockdown of HIF-1α could reduce glucose uptake and lactate production in both HCT116 and SW620 cells, which confirms the role of HIF-1α in mediating Warburg effect. However, matrine did not reduce either glucose uptake or lactate production in HIF-1α knockdown cells, indicating that matrine’s effect on reversing Warburg effect depends on HIF-1α activity. We then carried out MTT assay to examine matrine’s effect on cell growth in HIF-1α knockdown cells. Knockdown of HIF-1α decreased the growth rate of HCT116 and SW620 cells (**Figure S2C**), which confirm that HIF-1α could be a therapeutic target for decelerating colon cancer cell growth. Meanwhile, the growth inhibitory effect of matrine was largely attenuated in HIF-1α knockdown groups compared to the control group (**Figure 5C**), especially at high concentrations (6.0∼8.0 mM). These results suggested that the anticancer effect of matrine is in part, although not entirely, mediated by the inhibition of HIF-1α.

Furthermore, we examined whether overexpression of exogenous HIF-1α could rescue these two cells from matrine’s effect. qPCR and Western blot confirmed the overexpression of HIF-1α by transfecting pCMV5-HIF1A vectors (**Figures S2D, E**). Exogenous HIF-1α did not alter the growth rate of HCT116 and SW620 cells (**Figure S2F**). Matrine did not reduce glucose uptake and lactate production in HIF-1α overexpressed cells, indicating that exogenous HIF-1α could overcome matrine’s effect on reversing Warburg effect. Moreover, MTT assay shows that overexpression of HIF-1α could overcome a part of the toxicity of matrine on colon cancer cells (**Figure 5F**). This compensated effect of exogenous HIF-1α further confirmed the dependence of matrine’s cytotoxicity on the inhibition of HIF-1α.

## Discussion

The Warburg effect is the most distinguishing characteristic of energy metabolism in cancer cells. Multiple genes are involved in this complex controlled process. Modulating one gene may not be sufficient to suppress tumors and might even result in drug resistance. As a critical transcription factor, HIF-1 activated several glycolysis genes (e.g., GLUT1, GLUT4, HK1, HK2, LDHA), hence is an attractive target for modulating the Warburg effect. HIF-1 pathway is usually overactive in cancer cells, which is because of the hypoxic condition in the core of solid tumor as well as the activation of oncogene or loss of tumor suppressors. Besides glucose metabolism, the hyperactivation of HIF-1 pathway also triggers many other crucial cancer hallmarks such as angiogenesis, cell invasion, and metastasis, and has a vital role in tumor survival and progression (Semenza, 2002; Wilson and Hay, 2011). Therefore, HIF-1 has emerged as a promising anticancer therapeutic target (Li and Ye, 2010; Rey et al., 2017). In the present study, we identified matrine as a new HIF-1α inhibitor. Matrine inhibits the mRNA and protein expression of HIF-1α, thereby suppressing the transcription of GLUT1, HK2, and LDHA, the downstream targets of HIF-1, which are the key enzymes involved in the glycolytic energy metabolism of cancer cells. These effects lead to the suppression of the Warburg effect, i.e., decreasing glucose uptake and lactate production. Knockdown of HIF-1α or overexpression of HIF-1α could completely reverse matrine’s effect on glucose uptake and lactate production, indicating that the effect of matrine on Warburg effect depends on the inhibition of HIF-1α. It is worth to note that although Warburg effect provides a great advantage to the growth of cancer cells, ablation of Warburg effect can not entirely suppress tumor growth due to the metabolic plasticity of cancer cells (Marchiq et al., 2015; de Padua et al., 2017; Zdralevic et al., 2018a; Zdralevic et al., 2018b). Our results show that knockdown of HIF-1α or overexpression of HIF-1α largely attenuated the effect of matrine on cell growth, but not entirely as for the effect on glycolysis. This suggested that besides inhibiting HIF-1α and Warburg effect, other mechanisms may be involved in the growth inhibitory effect of matrine.

The growth inhibitory effect of matrine has been reported in a variety of cancers, including bladder cancer (Yang et al., 2017), breast cancer (Koh et al., 2008), myeloma (Zhou et al., 2015), lung cancer and hepatoma (Zhang et al., 2009), and also other colon cancer cells (Chang et al., 2013; Zhang et al., 2014). Consistent with these studies, our results show that matrine could suppress colon cancer cell growth. A possible mechanism that mediates matrine’s growth inhibitory effect is the suppression of the PI3K/AKT pathway (Li et al., 2012; Yang et al., 2017). PI3K/AKT pathway modulates mTOR and inhibit HIF-1α protein synthesis and thus reverse Warburg effect. Our data illustrate that matrine modulate the mRNA level of HIF-1α. Moreover, we also found that overexpressing exogenous HIF-1α could overcome a part of matrine’s cytotoxicity, which indicates that matrine may not affect exogenous HIF-1α. Therefore, our finding of matrine’s inhibitory effect on colon cancer cell growth may be independent of the PI3K/AKT/mTOR pathway. Matrine may suppress HIF-1α at transcription level. NF-κB is a critical transcriptional activator of HIF-1α (Rius et al., 2008), and the suppression effect of matrine on NF-κB pathway has also been reported (Niu et al., 2017). It is possible that the transcriptional inhibitory effect of matrine on HIF-1α is *via* the suppression of NF-κB pathway.

Besides growth inhibition, the cytotoxicity of matrine on cancer cells has also been reported. In our study, we found that a high concentration of matrine has significant toxicity on colon cancer cells (**Figure 1A**). Apoptosis is one of the most common death forms induced by matrine (Liu and Jiang, 2006; Luo et al., 2007; Zhang et al., 2010; Tan et al., 2013; Li et al., 2015; Gu et al., 2018). However, the mechanisms on how the apoptosis is triggered remain unknown. Current studies about matrine’s pro-apoptosis effect limited to that matrine could increase the expression of caspase-3, caspase-9 and some pro-apoptosis gene such as Bcl-2 family (Luo et al., 2007; Dai et al., 2009; Liang et al., 2012; Tan et al., 2013), without identifying the upstream targets. It is reported that HIF-1α has an anti-apoptosis effect on cancer cells. Nishimoto et al. reported that the activation of HIF-1α led to the acquisition of anti-apoptosis in human colon cancer cells (Nishimoto et al., 2014). Similar results were reported in A549 lung cancer cells, that HIF-1α protected A549 cells from drug-induced apoptosis (Schnitzer et al., 2006). Those anti-apoptosis effects were also reported in HUMEC cells (Yu et al., 2004), and HepG2 liver cancer cells (Piret et al., 2004). Our finding illustrated that matrine inhibited the transcription and protein expression of HIF-1α, which might also partially explain the pro-apoptosis effect of matrine.

Cellular regulation of HIF-1α is primarily at the level of protein degradation or protein translation. Therefore, most of the identified HIF-1α inhibitors target these pathways. Examples include Wortmannin and LY294002 as the PI3K/AKT inhibitors (Jiang et al., 2001), rapamycin and its chemical derivatives, CCI-779 which are mTOR inhibitors (Majumder et al., 2004), and geldanamycin that reduces heat shock protein 90 binding to HIF-1α to destabilize folding and increase proteasomal degradation (Mabjeesh et al., 2002). Other novel HIF-1 inhibitors that induce the degradation of HIF-1a protein have been identified, such as PX-478 and YC-1, but their precise mechanisms of action remain to be established (Koh et al., 2008; Tsui et al., 2012). However, it has also been suggested that, under hypoxic conditions, levels of HIF-1α mRNA may be a limiting factor affecting the rate of protein translation (Young et al., 2008). Since hypoxia is a common phenomenon in the core of solid tumor, our finding that matrine inhibit the mRNA level of HIF-1α would be more targeting the limiting factor and may be more utilized in solid tumor treatment.

One of the challenges for anticancer drug development is the reduction of side effect. The *in vivo* usage of matrine for cancer treatment in mice have been reported in several studies. The dose ranges from 25 to 100 mg/kg (Zhou et al., 2014; Wu et al., 2017; Cho et al., 2018; Shen et al., 2018; Wu et al., 2018). In our study, we use 80 mg/kg (0.32 mmol/kg) and the 7-days daily intraperitoneal administration shows distinct anticancer effect without significant side effects. All the mice were well tolerated without any sign of sickness. However, 7 days is quite a short-term course. Long-term exposure to matrine and closely examinations such as pathological sections for organ toxicity are required for the future development of matrine for clinical usage.

The present study has identified a new mechanisms of matrine’s anticancer effect. Our data illustrated that matrine could inhibit the transcription of HIF-1α and thus suppressed the HIF-1α-mediated Warburg effect. This finding provides a strong rationale for future clinical trials for the new application of matrine for colorectal cancer.

## Data Availability Statement

The datasets generated for this study are available on request to the corresponding author.

## Ethics Statement

The animal study was reviewed and approved by The Committee on the Ethics of Animal Experiments of Xiamen University.

## Author Contributions

XH, TH, GS, DW, and Y-YZ designed the study. XH, LZ, YX, KZ, JP, YL, YY, XX, PM, HC, and WZ performed the experiments. XH and Y-YZ wrote the manuscript. GS and DW contributed to manuscript revision. All authors read and approved the submitted version.

## Funding

The present study was supported by grants from the National Natural Science Foundation of China (No. 81602560 to XH and No. 81572589 to Y-YZ), grants from the Natural Science Foundation of Fujian Province (No. 2017J06020, 2017R1036-4 to Y-YZ, No.2019R1001-5 to TH and No. 2018R1036-5 to PM).

## Conflict of Interest

The authors declare that the research was conducted in the absence of any commercial or financial relationships that could be construed as a potential conflict of interest.

## References

[B1] Chang C. Liu S. P. Fang C. H. He R. S. Wang Z. Zhu Y. Q. (2013). Effects of matrine on the proliferation of HT29 human colon cancer cells and its antitumor mechanism. Oncol. Lett. 6 (3), 699–704. 10.3892/ol.2013.1449 24137393 PMC3789009

[B2] Chen Z. Lu W. Garcia-Prieto C. Huang P. (2007). The Warburg effect and its cancer therapeutic implications. J. Bioenerget. Biomembr. 39 (3), 267–274. 10.1007/s10863-007-9086-x 17551814

[B3] Cho Y. R. Lee J. H. Kim J. H. Lee S. Y. Yoo S. Jung M. K. (2018). Matrine suppresses KRAS-driven pancreatic cancer growth by inhibiting autophagy-mediated energy metabolism. Mol. Oncol. 12 (7), 1203–1215. 10.1002/1878-0261.12324 29791786 PMC6026868

[B4] Courtnay R. Ngo D. C. Malik N. Ververis K. Tortorella S. M. Karagiannis T. C. (2015). Cancer metabolism and the Warburg effect: the role of HIF-1 and PI3K. Mol. Biol. Rep. 42 (4), 841–851. 10.1007/s11033-015-3858-x 25689954

[B5] Dai Z. J. Gao J. Ji Z. Z. Wang X. J. Ren H. T. Liu X. X. (2009). Matrine induces apoptosis in gastric carcinoma cells via alteration of Fas/FasL and activation of caspase-3. J. Ethnopharmacol. 123 (1), 91–96. 10.1016/j.jep.2009.02.022 19429345

[B6] de Padua M. C. Delodi G. Vucetic M. Durivault J. Vial V. Bayer P. (2017). Disrupting glucose-6-phosphate isomerase fully suppresses the “Warburg effect” and activates OXPHOS with minimal impact on tumor growth except in hypoxia. Oncotarget 8 (50), 87623–87637. 10.18632/oncotarget.21007 29152106 PMC5675658

[B7] Denko N. C. (2008). Hypoxia, HIF1 and glucose metabolism in the solid tumour. Nat. Rev. Cancer 8 (9), 705–713. 10.1038/nrc2468 19143055

[B8] Gu Y. Y. Chen M. H. May B. H. Liao X. Z. Liu J. H. Tao L. T. (2018). Matrine induces apoptosis in multiple colorectal cancer cell lines in vitro and inhibits tumour growth with minimum side effects in vivo via Bcl-2 and caspase-3. Phytomedicine 51, 214–225. 10.1016/j.phymed.2018.10.004 30466620

[B9] Guo L. Xue T. Y. Xu W. Gao J. Z. (2013). Matrine promotes G0/G1 arrest and down-regulates cyclin D1 expression in human rhabdomyosarcoma cells. Panminerva Med. 55 (3), 291–296. 24088803

[B10] Jaakkola P. Mole D. R. Tian Y. M. Wilson M. I. Gielbert J. Gaskell S. J. (2001). Targeting of HIF-alpha to the von Hippel-Lindau ubiquitylation complex by O2-regulated prolyl hydroxylation. Science 292 (5516), 468–472. 10.1126/science.1059796 11292861

[B11] Jiang B. H. Jiang G. Zheng J. Z. Lu Z. Hunter T. Vogt P. K. (2001). Phosphatidylinositol 3-kinase signaling controls levels of hypoxia-inducible factor 1. Cell Growth Differ.: Mol. Biol. J. Am. Assoc. Cancer Res. 12 (7), 363–369. 11457733

[B12] Koh M. Y. Spivak-Kroizman T. Venturini S. Welsh S. Williams R. R. Kirkpatrick D. L. (2008). Molecular mechanisms for the activity of PX-478, an antitumor inhibitor of the hypoxia-inducible factor-1alpha. Mol. Cancer Ther. 7 (1), 90–100. 10.1158/1535-7163.MCT-07-0463 18202012

[B13] Li Y. Ye D. (2010). Cancer therapy by targeting hypoxia-inducible factor-1. Curr. Cancer Drug Targets 10 (7), 782–796. 10.2174/1568210205789830096 20578983

[B14] Li L. Q. Li X. L. Wang L. Du W. J. Guo R. Liang H. H. (2012). Matrine inhibits breast cancer growth via miR-21/PTEN/Akt pathway in MCF-7 cells. Cell. Physiol. Biochem.: Int. J. Exp. Cell. Physiol. Biochem. Pharmacol. 30 (3), 631–641. 10.1159/000341444 22832383

[B15] Li H. Li X. Bai M. Suo Y. Zhang G. Cao X. (2015). Matrine inhibited proliferation and increased apoptosis in human breast cancer MCF-7 cells via upregulation of Bax and downregulation of Bcl-2. Int. J. Clin. Exp. Pathol. 8 (11), 14793–14799. 26823806 PMC4713592

[B16] Liang C. Z. Zhang J. K. Shi Z. Liu B. Shen C. Q. Tao H. M. (2012). Matrine induces caspase-dependent apoptosis in human osteosarcoma cells in vitro and in vivo through the upregulation of Bax and Fas/FasL and downregulation of Bcl-2. Cancer Chemother. Pharmacol. 69 (2), 317–331. 10.1007/s00280-011-1699-4 21717192

[B17] Liu X. S. Jiang J. (2006). Molecular mechanism of matrine-induced apoptosis in leukemia K562 cells. Am. J. Chin. Med. 34 (6), 1095–1103. 10.1142/S0192415X06004557 17163597

[B18] Liu J. Zhu M. Shi R. Yang M. (2003). Radix Sophorae flavescentis for chronic hepatitis B: a systematic review of randomized trials. Am. J. Chin. Med. 31 (3), 337–354. 10.1142/S0192415X03001107 12943166

[B19] Liu Y. Xu Y. Ji W. Li X. Sun B. Gao Q. (2014). Anti-tumor activities of matrine and oxymatrine: literature review. Tumour Biol.: J. Int. Soc Oncodev. Biol. Med. 35 (6), 5111–5119. 10.1007/s13277-014-1680-z 24526416

[B20] Lu H. Forbes R. A. Verma A. (2002). Hypoxia-inducible factor 1 activation by aerobic glycolysis implicates the Warburg effect in carcinogenesis. J. Biol. Chem. 277 (26), 23111–23115. 10.1074/jbc.M202487200 11943784

[B21] Luo C. Zhu Y. Jiang T. Lu X. Zhang W. Jing Q. (2007). Matrine induced gastric cancer MKN45 cells apoptosis via increasing pro-apoptotic molecules of Bcl-2 family. Toxicology 229 (3), 245–252. 10.1016/j.tox.2006.10.020 17134813

[B22] Ma Y. Zou F. Xiong J. Wan W. Yin L. Li X. (2015). Effect of Matrine on HPAC cell migration by down-regulating the expression of MT1-MMP via Wnt signaling. Cancer Cell Int. 15, 59. 10.1186/s12935-015-0210-4 26113801 PMC4480578

[B23] Mabjeesh N. J. Post D. E. Willard M. T. Kaur B. Van Meir E. G. Simons J. W. (2002). Geldanamycin induces degradation of hypoxia-inducible factor 1alpha protein via the proteosome pathway in prostate cancer cells. Cancer Res. 62 (9), 2478–2482. 11980636

[B24] Majumder P. K. Febbo P. G. Bikoff R. Berger R. Xue Q. McMahon L. M. (2004). mTOR inhibition reverses Akt-dependent prostate intraepithelial neoplasia through regulation of apoptotic and HIF-1-dependent pathways. Nat. Med. 10 (6), 594–601. 10.1038/nm1052 15156201

[B25] Marchiq I. Le Floch R. Roux D. Simon M. P. Pouyssegur J. (2015). Genetic disruption of lactate/H+ symporters (MCTs) and their subunit CD147/BASIGIN sensitizes glycolytic tumor cells to phenformin. Cancer Res. 75 (1), 171–180. 10.1158/0008-5472.CAN-14-2260 25403912

[B26] Masoud G. N. Li W. (2015). HIF-1alpha pathway: role, regulation and intervention for cancer therapy. Acta Pharm. Sin. B. 5 (5), 378–389. 10.1016/j.apsb.2015.05.007 26579469 PMC4629436

[B27] Nishimoto A. Kugimiya N. Hosoyama T. Enoki T. Li T. S. Hamano K. (2014). HIF-1alpha activation under glucose deprivation plays a central role in the acquisition of anti-apoptosis in human colon cancer cells. Int. J. Oncol. 44 (6), 2077–2084. 10.3892/ijo.2014.2367 24718784

[B28] Niu Y. Dong Q. Li R. (2017). Matrine regulates Th1/Th2 cytokine responses in rheumatoid arthritis by attenuating the NF-kappaB signaling. Cell Biol. Int. 41 (6), 611–621. 10.1002/cbin.10763 28295853

[B29] Piret J. P. Lecocq C. Toffoli S. Ninane N. Raes M. Michiels C. (2004). Hypoxia and CoCl2 protect HepG2 cells against serum deprivation- and t-BHP-induced apoptosis: a possible anti-apoptotic role for HIF-1. Exp. Cell Res. 295 (2), 340–349. 10.1016/j.yexcr.2004.01.024 15093734

[B30] Rey S. Schito L. Wouters B. G. Eliasof S. Kerbel R. S. (2017). Targeting Hypoxia-Inducible Factors for Antiangiogenic Cancer Therapy. Trends Cancer 3 (7), 529–541. 10.1016/j.trecan.2017.05.002 28718406

[B31] Rius J. Guma M. Schachtrup C. Akassoglou K. Zinkernagel A. S. Nizet V. (2008). NF-kappaB links innate immunity to the hypoxic response through transcriptional regulation of HIF-1alpha. Nature 453 (7196), 807–811. 10.1038/nature06905 18432192 PMC2669289

[B32] Schnitzer S. E. Schmid T. Zhou J. Brune B. (2006). Hypoxia and HIF-1alpha protect A549 cells from drug-induced apoptosis. Cell Death Diff. 13 (9), 1611–1613. 10.1038/sj.cdd.4401864 16456580

[B33] Semenza G. L. (2002). HIF-1 and tumor progression: pathophysiology and therapeutics. Trends Mol. Med. 8 (4 Suppl), S62–S67. 10.1016/S1471-4914(02)02317-1 11927290

[B34] Semenza G. L. (2003). Targeting HIF-1 for cancer therapy. Nat. Rev. Cancer 3 (10), 721–732. 10.1038/nrc1187 13130303

[B35] Shen X. Huang J. Liu G. Zhang H. Zhang X. Kong X. (2018). Matrine inhibits neuroblastoma cell proliferation and migration by enhancing Tribbles 3 expression. Oncol. Res. 26 (7), 1133–1142. 10.3727/096504018X15168461629558 29386091 PMC7844772

[B36] Tan C. Qian X. Jia R. Wu M. Liang Z. (2013). Matrine induction of reactive oxygen species activates p38 leading to caspase-dependent cell apoptosis in non-small cell lung cancer cells. Oncol. Rep. 30 (5), 2529–2535. 10.3892/or.2013.2727 24026034

[B37] Tsui L. Fong T. H. Wang I. J. (2012). YC-1 targeting of hypoxia-inducible factor-1alpha reduces RGC-5 cell viability and inhibits cell proliferation. Mol. Vision 18, 1594–1603. PMC3380911 22736948

[B38] Vander Heiden M. G. Cantley L. C. Thompson C. B. (2009). Understanding the Warburg effect: the metabolic requirements of cell proliferation. Science 324 (5930), 1029–1033. 10.1126/science.1160809 19460998 PMC2849637

[B39] Warburg O. (1956). On the origin of cancer cells. Science 123 (3191), 309–314. 10.1126/science.123.3191.309 13298683

[B40] Wilson W. R. Hay M. P. (2011). Targeting hypoxia in cancer therapy. Nat. Rev. Cancer 11 (6), 393–410. 10.1038/nrc3064 21606941

[B41] Wu J. Hu G. Dong Y. Ma R. Yu Z. Jiang S. (2017). Matrine induces Akt/mTOR signalling inhibition-mediated autophagy and apoptosis in acute myeloid leukaemia cells. J. Cell Mol. Med. 21 (6), 1171–1181. 10.1111/jcmm.13049 28026112 PMC5431164

[B42] Wu D. Shao K. Zhou Q. Sun J. Wang Z. Yan F. (2018). Autophagy and ubiquitin-mediated proteolytic degradation of pml/raralpha fusion protein in matrine-induced differentiation sensitivity recovery of ATRA-resistant APL (NB4-LR1) cells: *in Vitro* and *in Vivo* studies. Cell Physiol. Biochem. 48 (6), 2286–2301. 10.1159/000492646 30114705

[B43] Xu B. Xu M. Tian Y. Yu Q. Zhao Y. Chen X. (2017). Matrine induces RIP3-dependent necroptosis in cholangiocarcinoma cells. Cell Death Discovery 3, 16096. 10.1038/cddiscovery.2016.96 28179994 PMC5253620

[B44] Yang Y. Guo J. X. Shao Z. Q. Gao J. P. (2017). Matrine inhibits bladder cancer cell growth and invasion in vitro through PI3K/AKT signaling pathway: An experimental study. Asian Pac. J. Trop. Med. 10 (5), 515–519. 10.1016/j.apjtm.2017.05.009 28647190

[B45] Young R. M. Wang S. J. Gordan J. D. Ji X. Liebhaber S. A. Simon M. C. (2008). Hypoxia-mediated selective mRNA translation by an internal ribosome entry site-independent mechanism. J. Biol. Chem. 283 (24), 16309–16319. 10.1074/jbc.M710079200 18430730 PMC2423241

[B46] Yu E. Z. Li Y. Y. Liu X. H. Kagan E. McCarron R. M. (2004). Antiapoptotic action of hypoxia-inducible factor-1 alpha in human endothelial cells. Lab. Invest. J. Tech. Methods Pathol. 84 (5), 553–561. 10.1038/labinvest.3700071 15064771

[B47] Zdralevic M. Brand A. Di Ianni L. Dettmer K. Reinders J. Singer K. (2018a). Double genetic disruption of lactate dehydrogenases A and B is required to ablate the “Warburg effect” restricting tumor growth to oxidative metabolism. J. Biol. Chem. 293 (41), 15947–15961. 10.1074/jbc.RA118.004180 30158244 PMC6187639

[B48] Zdralevic M. Vucetic M. Daher B. Marchiq I. Parks S. K. Pouyssegur J. (2018b). Disrupting the ‘Warburg effect’ re-routes cancer cells to OXPHOS offering a vulnerability point via ‘ferroptosis’-induced cell death. Adv. Biol. Regul. 68, 55–63. 10.1016/j.jbior.2017.12.002 29306548

[B49] Zhang Y. Zhang H. Yu P. Liu Q. Liu K. Duan H. (2009). Effects of matrine against the growth of human lung cancer and hepatoma cells as well as lung cancer cell migration. Cytotechnology 59 (3), 191–200. 10.1007/s10616-009-9211-2 19649719 PMC2774570

[B50] Zhang J. Q. Li Y. M. Liu T. He W. T. Chen Y. T. Chen X. H. (2010). Antitumor effect of matrine in human hepatoma G2 cells by inducing apoptosis and autophagy. World J. gastroenterol. 16 (34), 4281–4290. 10.3748/wjg.v16.i34.4281 20818811 PMC2937108

[B51] Zhang S. Cheng B. Li H. Xu W. Zhai B. Pan S. (2014). Matrine inhibits proliferation and induces apoptosis of human colon cancer LoVo cells by inactivating Akt pathway. Mol. Biol. Rep. 41 (4), 2101–2108. 10.1007/s11033-014-3059-z 24452711

[B52] Zhou H. Xu M. Gao Y. Deng Z. Cao H. Zhang W. (2014). Matrine induces caspase-independent program cell death in hepatocellular carcinoma through bid-mediated nuclear translocation of apoptosis inducing factor. Mol. Cancer 13, 59. 10.1186/1476-4598-13-59 24628719 PMC4007561

[B53] Zhou Y. H. Feng J. Y. You L. S. Meng H. T. Qian W. B. (2015). Matrine and CYC116 synergistically inhibit growth and induce apoptosis in multiple myeloma cells. Chin. J. Integr. Med. 21 (8), 635–639. 10.1007/s11655-015-1975-y 25804197

[B54] Zou C. Wang Y. Shen Z. (2005). 2-NBDG as a fluorescent indicator for direct glucose uptake measurement. J. Biochem. Biophys. Methods 64 (3), 207–215. 10.1016/j.jbbm.2005.08.001 16182371

