# Supplementary figures and images for: Matrine Reverses the Warburg Effect and Suppresses Colon Cancer Cell Growth via Negatively Regulating HIF-1α
Source: Front Pharmacol. 2019 Nov 28;10:1437. doi: 10.3389/fphar.2019.01437 (PMC6892950; doi:10.3389/fphar.2019.01437)

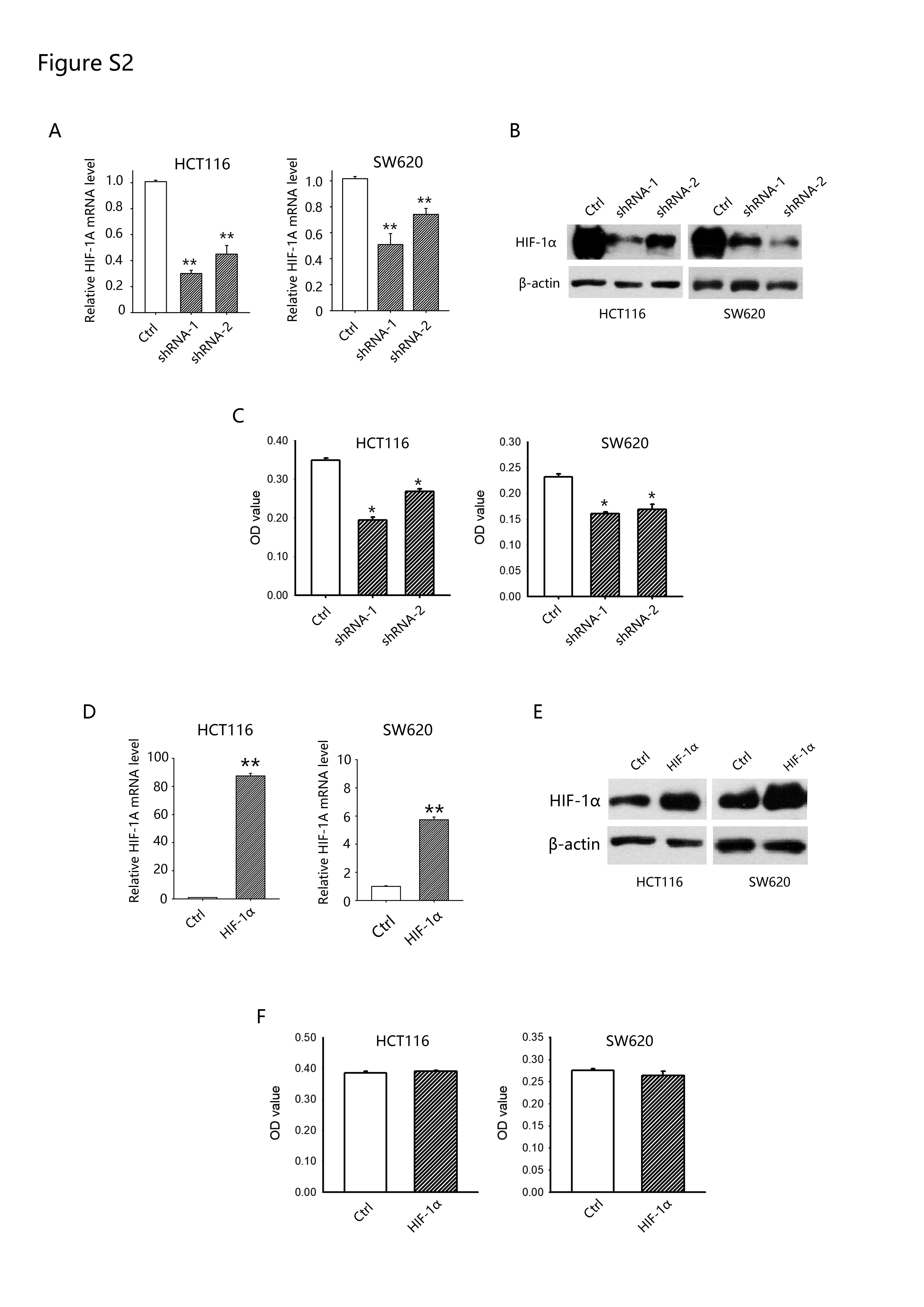

Supplement: Supplementary file 1 [file Image_1.tif]

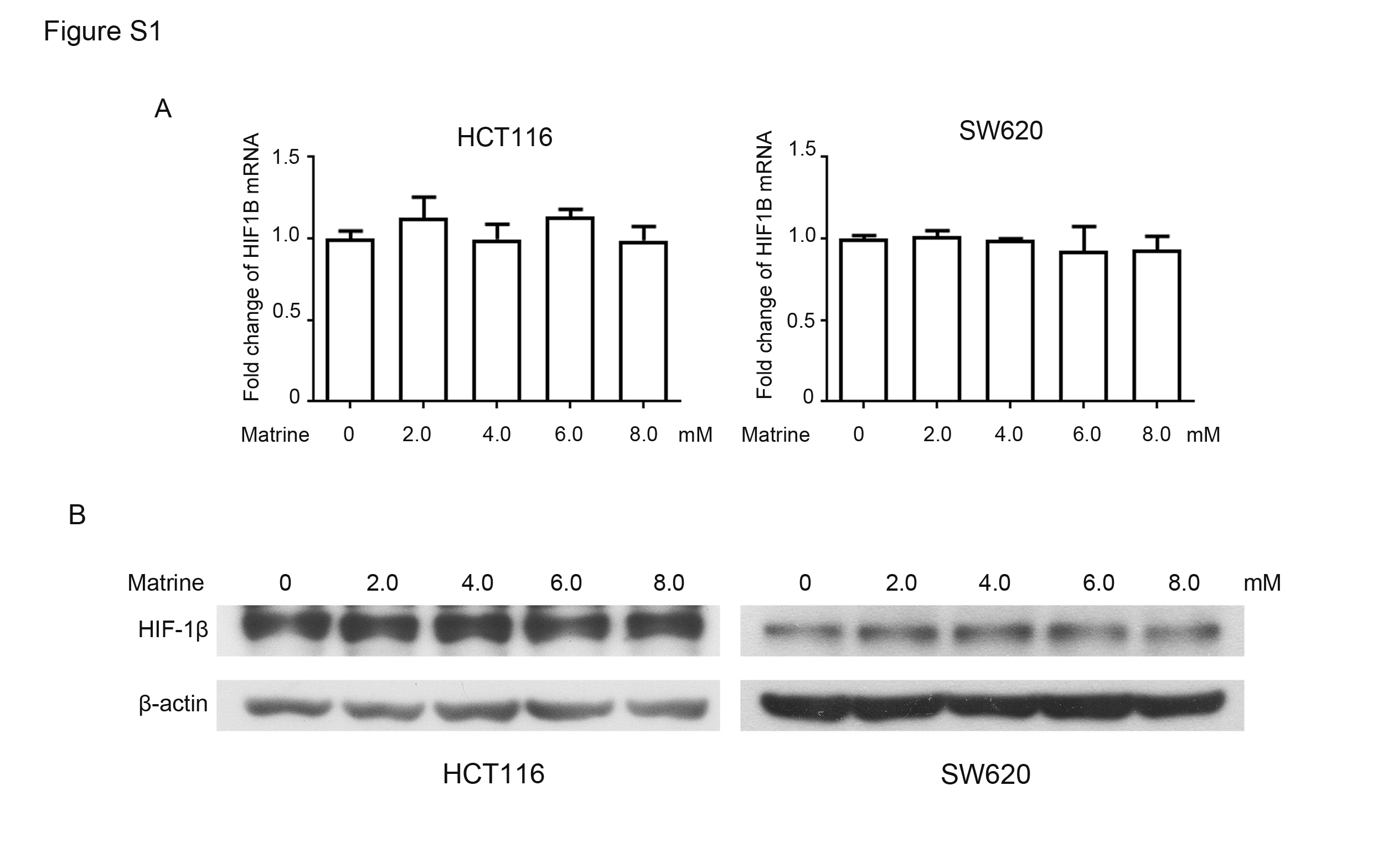

Supplement: Supplementary file 2 [file Image_2.tif]
