# Supplementary material for: Matrine Reverses the Warburg Effect and Suppresses Colon Cancer Cell Growth via Negatively Regulating HIF-1α
Source: Front Pharmacol. 2019 Nov 28;10:1437. doi: 10.3389/fphar.2019.01437 (PMC6892950; doi:10.3389/fphar.2019.01437)
Supplement: Supplementary file 3 [file Table_1.docx]

Supplemental figure legends

Figure S1

(A) HIF-1β mRNA level as quantified by qRT-PCR in HCT116 and SW620 cells with the treatment of 0~8.0 mM matrine for 12 hours. Data were expressed as mean ± SEM. (B) Western blotting shows the protein expression of HIF-1β in HCT116 and SW620 cells with 12 hours treatment of 0~8.0 mM matrine.

Figure S2

(A) qRT-PCR analysis of HIF-1α validated the knockdown effect of shRNA. Data are expressed as mean ± SEM, n=3. **, P < 0.01. (B) Western blotting showed the knockdown effect of HIF-1α by shRNA in protein expression level. (C) MTT assay shows the effect of HIF-1α knockdown on cell viability. Data are expressed as mean ± SEM, n=3. *, P < 0.05. (D) qRT-PCR analysis to validate the overexpression of HIF-1α. Data are expressed as mean ± SEM, n=3. **, P < 0.01. (E) Western blotting showed the overexpression of HIF-1α. (C) MTT assay shows the effect of HIF-1α overexpression on cell viability. Data are expressed as mean ± SEM, n=3.
